# Awareness and Perception of Myalgic Encephalomyelitis and Chronic Fatigue Syndrome Among Pain Specialists: A Questionnaire-Based Study

**DOI:** 10.7759/cureus.81030

**Published:** 2025-03-23

**Authors:** Gürsan Güneş Uygun, Salim Taner Gözükızıl, Ayşegül Bilen

**Affiliations:** 1 Department of Pain Medicine, University of Health Sciences, Prof. Dr. Cemil Taşcıoğlu City Hospital, Istanbul, TUR

**Keywords:** chronic fatigue syndrome, dysautonomia, myalgic encephalomyelitis, pain medicine, post-exertional malaise (pem)

## Abstract

Background: This study aims to explore the perceptions and clinical approaches of pain management specialists toward myalgic encephalomyelitis/chronic fatigue syndrome (ME/CFS), a condition characterized by persistent fatigue, widespread pain, unrefreshing sleep, and autonomic dysfunction. Given the challenges in diagnosis and management, pain specialists may play a pivotal role in symptom relief. By identifying variations in evaluation and treatment practices, this study seeks to enhance the recognition of ME/CFS and improve its clinical management within pain medicine.

Material and methods: The questionnaire was distributed in printed form to 250 pain specialists in Turkey. Given the limited number of pain physicians in the country, the study aimed to encompass all actively practicing specialists. The survey included four demographic questions, eight true-false questions, 12 multiple-choice questions, and four rating-scale questions. Data were collected anonymously. Data were analyzed using Statistical Product and Service Solutions (SPSS, version 27.0; IBM SPSS Statistics for Windows, Armonk, NY), with descriptive statistics and chi-square (χ²) tests applied to examine the relationships between awareness levels and attitudinal variables.

Results: In Turkey, 106 pain medicine physicians (42.4%) participated in the study. The average age was 40.6±8.52 years. Among the participants, 39.6% had previously heard of myalgic encephalomyelitis. Physicians were inclined to first ask the question, "Do you think you get enough sleep at night?" when evaluating these patients, with a rate of 63.2%. The majority of participants (65.9%) stated that they "occasionally" or "rarely" considered the relationship between fatigue and orthostatic intolerance. Additionally, 37.7% believed that this disease is a subtype of depression. The statement, "chronic fatigue decreases with intense aerobic exercise," was agreed upon by 50.9% of participants. This controversial statement was particularly more common among those who were unaware of ME/CFS's alternative name (p=0.009) and those who did not take dysautonomic disorders into account (p=0.048). When considering an ME/CFS diagnosis, physicians most frequently referred patients to the physical medicine and rehabilitation department (32.1%). Those who preferred not to refer patients to any department (12.3%) tended to find it appropriate for a patient to seek consultation at a pain medicine clinic due to widespread body pain and fatigue (χ2=11.405, p=0.044).

Conclusion: This study is the first questionnaire-based research assessing pain physicians' awareness and attitudes toward ME/CFS. By highlighting their perspectives on its evaluation and management, our findings may improve recognition and clinical approaches to ME/CFS. Future research should focus on education and standardized guidelines to enhance patient care.

## Introduction

Myalgic encephalomyelitis/chronic fatigue syndrome (ME/CFS) is a complex condition that has long been debated in the medical literature and is typically classified among disorders commonly referred to as medically unexplained syndromes/symptoms. ME/CFS is primarily characterized by persistent and unexplained fatigue that is not alleviated by rest, non-restorative sleep, widespread pain, cognitive dysfunction, and post-exertional malaise (PEM) [1]. Due to its broad and heterogeneous symptom profile, diagnosing ME/CFS remains highly challenging as there are often no specific abnormalities detected in standard laboratory tests. Globally, there is no consensus regarding the optimal diagnostic approach or treatment for ME/CFS, further complicating clinical management.

One of the most comprehensive epidemiological studies on ME/CFS, conducted using data from the 2021-2022 National Health Interview Survey (NHIS), estimated that 1.3% of adults in the United States are affected by the condition. Despite its significant impact, more than 90% of individuals with ME/CFS remain undiagnosed [2]. The economic burden of ME/CFS is substantial, costing the US economy between $18 and $51 billion annually due to healthcare expenses and lost productivity. This considerable financial strain is primarily attributed to several key factors: (i) delayed diagnosis, leading to unnecessary medical expenditures and ineffective treatments; (ii) the inability of many patients to work or maintain full-time employment, resulting in significant income loss; and (iii) frequent doctor visits and referrals to multiple specialists due to the lack of a standardized diagnostic process [3].

While interdisciplinary collaboration is recommended for patient care, significant gaps exist in the integration of pain medicine into ME/CFS treatment frameworks. Pain physicians, given their expertise in pain modulation and management, can play a crucial role in symptom relief and improving the quality of life in ME/CFS patients. However, data regarding the perspectives and involvement of pain specialists in ME/CFS diagnosis and treatment are scarce in the literature, highlighting a critical gap in current research. Understanding the perspectives of pain physicians on ME/CFS could provide valuable insights for developing more effective and multidisciplinary treatment strategies. Given the substantial burden of pain and fatigue in these patients, a comprehensive, multidisciplinary approach that includes pain medicine, neurology, psychiatry, physical therapy, and immunology is essential. This study aims to evaluate the current clinical approaches and perspectives on ME/CFS among pain physicians in Turkey, contributing to the enhancement of pain management strategies and fostering interdisciplinary collaboration in ME/CFS care.

## Materials and methods

Ethical approval for this study was granted by the Istanbul Prof. Dr. Cemil Taşcıoğlu City Hospital Ethics Committee (protocol no: 2025/26). The official approval document can be found in the Appendices section. The questionnaire used in this study was newly developed, and no prior scale had been established on this topic. The questions were carefully designed based on relevant literature and expert opinions to ensure their appropriateness for the study objectives. A printed questionnaire was used as the data collection instrument and was distributed to pain physicians in training as well as actively practicing pain specialists. Informed consent was obtained from all participants, and responses were collected anonymously. The questionnaire comprised 28 questions in total, including four demographic questions, eight true/false items, 12 multiple-choice questions, and four questions answered using a rating scale. It was designed to comprehensively assess participants’ knowledge, awareness, and perceptions (Table 1).

**Table 1:** Questionnaire form.

Questionnaire					
Demographic Information					
Age	Gender	Hospital of Employment		Duration of Experience in the Field of Pain Medicine	
Assessment of the Accuracy of Statements					
Statements				True	False
ME/CFS is a disease that is frequently seen in older adults.					
Persistent fatigue that does not improve with rest is a significant symptom of ME/CFS.					
ME/CFS only presents with physical fatigue symptoms; mental fatigue is not observed.					
ME/CFS is considered a subtype of depression.					
Chronic fatigue decreases with intense aerobic exercise.					
ME/CFS is a disease that resolves within a few months.					
ME/CFS does not cause any problems in sexual life.					
Individuals with ME/CFS do not experience changes in their eating habits.					
Multiple-Choice Questions					
No answer choices are provided. Only the questions are listed.					
Do you find it appropriate for a patient to visit the pain clinic due to widespread body pain and fatigue?					
When questioning a patient presenting with fatigue, which of the following statements are you most likely to use first?					
Do you tend to think that fatigue complaints are caused by psychological factors?					
What is the first test you evaluate in patients presenting with fatigue?					
Have you heard of Myalgic Encephalomyelitis (ME) before?					
Did you know that ME is also referred to as ‘Chronic Fatigue Syndrome (CFS)’?					
Did you know that ME/CFS is classified under ICD code G93.3?					
Which symptoms do you think are the most common in ME/CFS?					
How severe do you think the pain associated with ME/CFS is?					
Which department would you prefer to refer a patient to if you strongly suspect ME/CFS?					
What is the biggest challenge you face when diagnosing ME/CFS?					
Would you find training on ME/CFS beneficial?					
Evaluation Using a Rating Scale					
Question	Always	Mostly	Occasionally	Rarely	Never
Do you inquire about the relationship between exercise and widespread body pain in your patients?					
Do you routinely ask about sleep patterns in patients presenting with widespread pain?					
In patients with fatigue complaints, do you tend to consider a dysautonomic disorder (e.g., orthostatic intolerance) as a possible cause?					
Do you tend to think that the fatigue complaints of patients presenting with fatigue are caused by a psychological condition?					

Data were analyzed using Statistical Product and Service Solutions (SPSS, version 27.0; IBM SPSS Statistics for Windows, Armonk, NY). Descriptive statistics were calculated, and the chi-square test of independence was applied to evaluate relationships between awareness levels and attitude variables. A p-value of <0.05 was considered statistically significant.

## Results

Demographic characteristics

**Figure 1:**
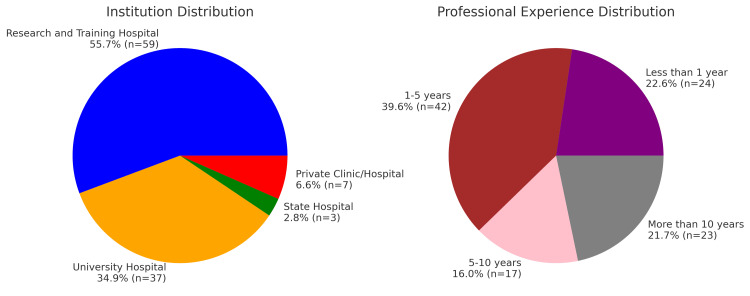
Demographic data.

A total of 106 physicians, including those currently undergoing training in pain medicine and those actively working as pain specialists, participated in our study. This corresponds to a participation rate of 42.4% among a total of 250 physicians. The gender distribution of the participating physicians was equal, with both female and male participants comprising 50.0% each (n=53). Figure 1 summarizes the demographic characteristics of the participants, including the type of institution where they work and their duration of professional experience. The mean age of the participants was 40.6±8.52 years, with an age range of 30-64 years.

Physicians’ inquiry patterns in the clinical assessment of fatigue and pain

A total of 73.6% of participants considered it appropriate for a patient to seek consultation at a pain clinic due to widespread body pain and fatigue. Among the participating physicians, the primary diagnostic evaluation for patients presenting with fatigue was a complete blood count and biochemical panel, selected by 73.6% (n=78) of respondents. Thyroid function tests and vitamin level assessments were each chosen by 12.3% (n=13) of participants. In contrast, rheumatic biomarkers and sleep studies were rarely considered as initial diagnostic tests, with only 0.9% (n=1) of physicians selecting each.

**Figure 2:**
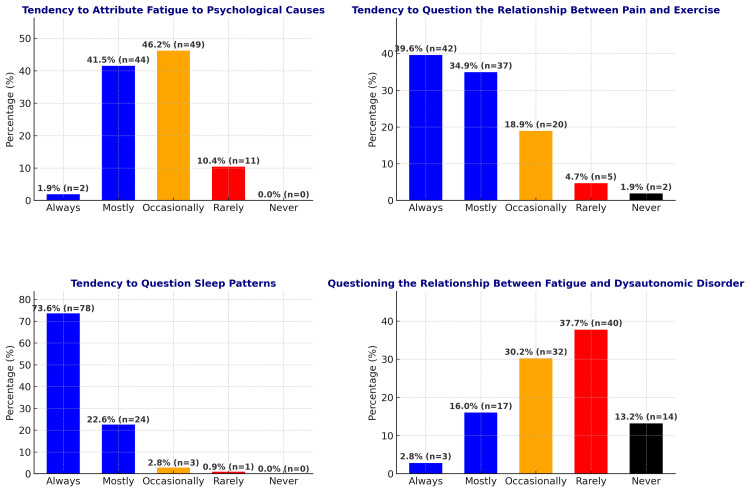
Evaluation of physicians' inquiry tendencies regarding various factors when assessing fatigue and widespread pain complaints.

In our study, we investigated the first questions that physicians typically ask patients presenting with fatigue complaints. The most frequently asked question, posed by 63.7% of the participants, was "Do you think you are getting enough sleep at night?". Subsequently, the tendencies to inquire about various factors while evaluating complaints of fatigue and widespread pain are illustrated in Figure 2. Figure 2 presents, through four separate graphs, the frequencies with which these complaints are associated with psychological factors, exercise, sleep patterns, and dysautonomic disorders.

Physicians' awareness of ME/CFS and its terminology

**Figure 3:**
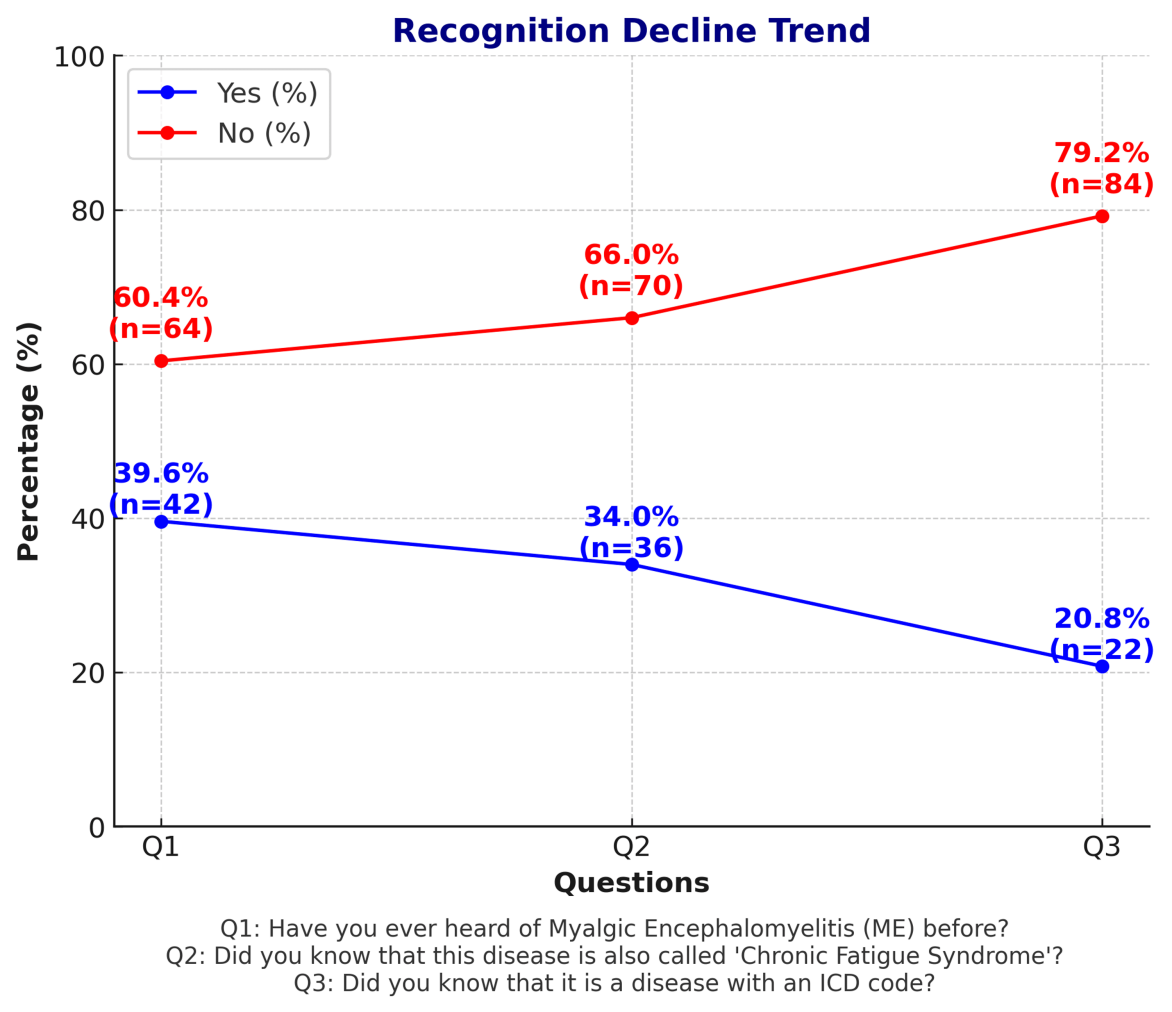
Trend of declining awareness.

Figure 3 presents the distribution of responses to the yes/no questions designed to assess the level of awareness regarding myalgic encephalomyelitis (ME).

Differential diagnosis of widespread pain and symptom recognition in ME/CFS

**Figure 4:**
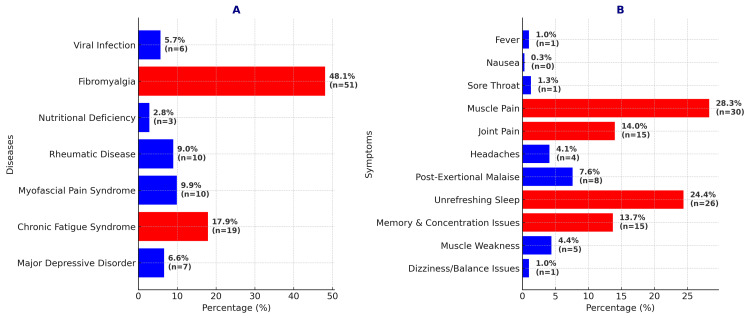
Physicians' assessments of widespread pain and myalgic encephalomyelitis/chronic fatigue syndrome (ME/CFS) symptoms. Chart A illustrates the differential diagnoses that physicians take into account for widespread body pain. Chart B highlights the symptoms most commonly linked to ME/CFS by physicians.

Following these evaluations, the differential diagnostic approaches for widespread pain and the level of awareness regarding ME/CFS symptoms among physicians are shown in Figure 4. Chart A displays the differential diagnoses considered by physicians for widespread body pain, while Chart B shows the symptoms they most frequently associate with ME/CFS. Notably, Chart B reflects the physicians' thoughts on ME/CFS, and these data do not represent actual epidemiological findings. In regards to the pain within the ME/CFS syndrome, 69.8% of the participants rated it as moderate, 23.6% as severe, and only 6.6% considered it to be mild.

Perception and misunderstandings of ME/CFS

**Figure 5:**
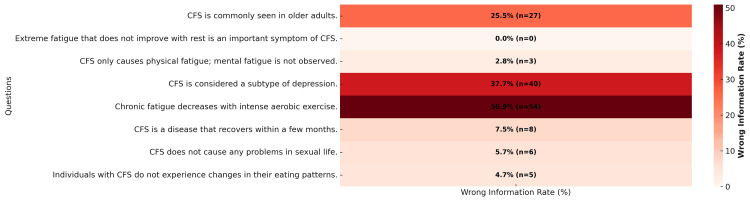
Physicians' knowledge level on chronic fatigue syndrome (CFS): heat map.

Furthermore, the level of knowledge regarding ME/CFS and common beliefs about this condition were also evaluated. Figure 5 visualizes the physicians’ knowledge levels on various topics related to ME/CFS using a heat map. This analysis helps to pinpoint the areas where differences in knowledge are most pronounced.

Associations between awareness, perceptions, and medical decisions

In our analysis, the most controversial statement was "chronic fatigue decreases with intense aerobic exercise" (Figure 5). Additionally, 50.9% of the participants answered "true" to this statement, indicating an incorrect evaluation. In this context, we examined which groups or individuals - with specific awareness and attitude characteristics - believed that chronic fatigue could be alleviated by intense aerobic exercise. Chi-square analysis revealed statistically significant differences. The results indicate that this belief is particularly prevalent among those who do not tend to consider that it may be associated with a dysautonomic disorder (chi-square=9.567, p=0.048) and among those who are unaware that another name for ME is "chronic fatigue syndrome" (chi-square=6.765, p=0.009) (Table 2).

**Table 2:** Evaluation of the tendency of participants who believe that chronic fatigue can be reduced with intensive aerobic exercise to associate it with dysautonomic disorders and their awareness of the alternative name for myalgic encephalomyelitis.

Item			Are you inclined to consider that fatigue complaints in your patients are related to a dysautonomia disorder?					Did you know that the alternative name for myalgic encephalomyelitis is "chronic fatigue syndrome"?	
			Always	Mostly	Occasionally	Rarely	Never	Yes	No
Chronic fatigue can be reduced with intensive aerobic exercise.	True	%(n)	5.6% (3)	16.7% (9)	18.5% (10)	42%(23)	16.7% (9)	22.2% (12)	77.8% (42)
	False	%(n)	0% (0)	15.4% (8)	42.3% (22)	32.7%(17)	9.6% (5)	46.2% (24)	53.8% (28)
Total		% (n)	2.8% (3)	16% (17)	30.2% (32)	37.7%(40)	13.2%(14)	34% (36)	66% (70)
Test value			χ²=9.567, p=0.048					χ²=6.765, p=0.009	

Another controversial statement was "ME/CFS is considered a subtype of depression." We examined whether this view was associated with physicians' approaches to evaluating fatigue and pain complaints. The relationship between the opinions linking ME/CFS with depression and the tendency to attribute fatigue complaints in patients to psychological causes is presented in Table 3. A chi-square test was performed to determine the statistical significance of the relationship between these two variables, and a significant association was found (chi-square=8.018, p=0.046). The results indicate that physicians who consider ME/CFS as a subtype of depression are more likely to attribute fatigue complaints to psychological causes.

**Table 3:** Evaluation of the relationship between opinions on whether depression is a subtype and the tendency to attribute the complaints of fatigue in patients presenting with this symptom to a psychological condition.

Item			Are you inclined to think that the fatigue complaints of patients presenting with this symptom are due to a psychological condition?			
			Always	Mostly	Occasionally	Rarely
ME/CFS is considered a subtype of depression.	True	n	2	21	15	2
		%	5%	52.5%	37.5%	5%
	False	n	0	23	34	9
		%	0%	34.8%	51.5%	13.6%
Total		n	2	44	49	11
		%	1.9%	41.5%	46.2%	10.4%
Test value		χ²=8.018, p=0.046				

No statistically significant relationship was found between the tendency to attribute fatigue complaints to a psychological condition and referral to psychiatry (chi-square=11.807, p=0.694). The relationship between the appropriateness of referring patients with widespread body pain and fatigue complaints to the pain clinic and the specialty areas to which clinicians refer when they strongly suspect an ME/CFS diagnosis was also evaluated (Table 4). The findings indicate that referral preferences are associated with opinions on the suitability of referrals to the pain clinic. Those who do not prefer referring patients to another clinic tend to consider that it is appropriate for a patient with widespread body pain and fatigue to visit a pain clinic (chi-square=11.405, p=0.044).

**Table 4:** Evaluation of the appropriateness of referring a patient to an pain clinic due to widespread body pain and fatigue and the relationship between the clinics to which patients are referred when ME/CFS is considered a strong possibility.

Item			Do you consider it appropriate for a patient to visit an pain clinic due to widespread body pain and fatigue?	
			Yes	No
Which clinic do you prefer to refer patients to when you consider myalgic encephalomyelitis/chronic fatigue syndrome a strong possibility?	Internal Medicine	n	5	3
		%	62.5%	37.5%
	Physical Therapy and Rehabilitation	n	20	14
		%	58.8%	41.2%
	Rheumatology	n	6	0
		%	100%	0%
	Psychiatry	n	18	5
		%	78.3%	21.7%
	Neurology	n	16	6
		%	72.7%	27.3%
	I prefer not to refer to another clinic.	n	13	0
		%	100%	0%
Total		n	78	28
		%	73.6%	26.4%
Test value		χ²=11.405, p=0.044		

Diagnostic challenges and perspectives on ME/CFS education

Furthermore, the greatest challenge in diagnosing ME/CFS was identified as the inadequacy of diagnostic tests (30.2%), followed by patients’ inability to clearly articulate their symptoms (24.5%), and the ambiguity of the clinical criteria (22.6%). The vast majority of participating physicians (96.2%, n=102) stated that they found education on ME/CFS to be beneficial. In contrast, only 3.8% (n=4) did not consider such education necessary.

## Discussion

In conditions such as CFS, which symptomatically overlaps with other chronic pain syndromes but may differ in their underlying pathophysiological mechanisms, a detailed evaluation of fatigue and pain processes is crucial for an accurate diagnosis. An accurate diagnosis directly influences the treatment plan and management strategies, thereby impacting the patient’s quality of life. Particularly among patients with similar symptom profiles, a thorough and meticulous assessment plays a fundamental role in personalizing treatment approaches.

Despite its significant impact on quality of life, there is a paucity of literature addressing clinicians' awareness and perception of this disorder. Our study addresses this gap by presenting the first questionnaire-based study on ME/CFS in the literature and, importantly, the first to assess physicians’ awareness and clinical approach to the condition.

When analyzing the gender distribution of the 106 physicians who participated in the study, the equal number of female and male participants indicates that the study provides a balanced sample in terms of gender representation. As shown in Figure 2, the institutional distribution revealed that the highest proportion (55.7%) was associated with training and research hospitals, followed by 34.9% with university hospitals, and 9.4% with other healthcare institutions (state hospitals and private hospitals/clinics). This distribution demonstrates that the study predominantly encompasses healthcare professionals working in academic and research-oriented healthcare institutions.

When evaluating physicians' tendency to associate fatigue with psychological causes, the high proportion (87.7%) of those who take psychological factors into account either "mostly" or "occasionally" indicates that physicians tend to consider psychosocial components when assessing such symptoms. In contrast, the fact that only 10.4% rarely make this connection suggests that some physicians place greater emphasis on biological or other physiological causes. In pain management, nonsteroidal anti-inflammatory drugs and opioids sometimes provide only limited relief. One reason for this limited efficacy may be that psychosocial factors - such as depression, which can exacerbate pain and its effects - and patient heterogeneity are not adequately considered. Therefore, to develop treatments tailored to individual needs, the variability in patients' pain experiences should be taken into account. 

When examining the initial question posed to patients presenting with fatigue complaints, it was observed that the majority of participants (63.2%) preferred the question, "Do you think you get sufficient sleep at night?" This distribution suggests that physicians tend to focus primarily on sleep patterns when evaluating patients with fatigue complaints. In our study, 73.6% of participants answered "Always" to the question regarding the frequency with which they assess sleep patterns in patients presenting with pain, indicating a high level of awareness among clinicians regarding the importance of sleep in pain management (Figure 3). Studies in the literature support the underlying biological mechanisms of this phenomenon. For example, a study by Djordjic et al. involving patients with chronic low back pain found that levels of neurofilament polypeptide, neuron-specific enolase, and protein S100B, which are biomarkers indicative of structural damage in nervous tissue, were significantly higher in patients experiencing sleep disturbances compared to those without any disturbances [4]. Additionally, a systematic review demonstrated that improvements in sleep quality among patients with chronic low back pain may be associated with reductions in pain and disability levels [5]. This finding underscores that treatments targeting sleep disturbances should be considered an integral component of pain management strategies.

A total of 65.9% of participants indicated that they consider the association between fatigue and orthostatic intolerance either "Occasionally" or "Rarely" (Figure 3). In orthostatic intolerance syndrome, due to sympathetic dysfunction in the vascular structures of the lower extremities, blood pools in these regions upon standing. This can lead to muscle pain and weakness as a result of insufficient vascular perfusion in the lower extremities [6]. A reduced amount of blood returning to the heart and reflex sympathetic discharge triggered by cerebral hypoperfusion may result in excessive activation of the sympathetic nervous system [4]. It has been suggested that the hyper noradrenergic state that develops in this process is associated with hypersensitivity of β-adrenergic receptors, which may increase energy consumption and lead to prolonged fatigue [7].

One possible reason for the low awareness of the link between fatigue and orthostatic intolerance is the difficulty in accessing evaluation methods. Participants who do not have access to these tests in clinical practice may fail to recognize the relationship between fatigue and dysautonomic disorders. Orthostatic intolerance is an objectively measurable symptom. Evaluation methods include the tilt table test and sudomotor tests, among which is the NASA Lean Test, developed by the Naval Aerospace Medical Research Laboratory (NASMRL) [8,9]. Increasing access to these methods could enhance clinical awareness and promote more frequent evaluation of this association.

Among the participating physicians, 39.6% reported having previously heard of ME, while the proportion who knew that the disease is also referred to as CFS declined to 34.0%. More specifically, awareness of the disease’s ICD code further decreased to 20.8% (Figure 4). These findings suggest that, while general awareness of the disease exists, knowledge gaps widen when it comes to terminology and specific medical details. The observed decline in awareness may be attributable to the diagnostic and pathophysiological ambiguity of ME/CFS, its terminological complexity, and its infrequent occurrence in clinical practice. This underscores the need for more comprehensive education and awareness initiatives.

Participants primarily considered CFS and fibromyalgia (FM) in patients presenting with widespread body pain and fatigue (Figure 5). In FM diagnosis, the presence of widespread pain throughout the body is a fundamental criterion [10]. In CFS, fatigue is the predominant symptom. It is important to assess the reduction in activity across various aspects of the patient's life (e.g., work, school, social life). For diagnosis, the specified thresholds for significant reduction must be met. CFS must persist for at least six months and is typically accompanied by profound fatigue. Additionally, post-exertional malaise (PEM), characterized by a decline in physical or cognitive function, must be present. Furthermore, patients' sleep should be non-restorative. Lastly, at least one additional symptom must be present for diagnosis; these may include cognitive impairment or orthostatic intolerance. In CFS, the frequency and severity of the symptoms must be taken into account to ensure diagnostic accuracy. If patients do not experience the specified symptoms at least half of the time and at a moderate to severe intensity, the ME/CFS diagnosis should be reconsidered [1]. Additionally, some biochemical differences have been identified between FM and CFS. While an increase in substance P is observed in the cerebrospinal fluid of FM patients, such an increase is not observed in CFS [11].

In our survey, the most controversial statement was "chronic fatigue decreases with intense aerobic exercise" (Figure 6). One of the diagnostic criteria for CFS/ME is PEM, which is characterized by the worsening of symptoms following physical or cognitive exertion. The underlying causes of this phenomenon include post-infectious dysregulation of the immune system, mitochondrial dysfunction, and disturbances in nervous system regulation [12]. Approaches such as graded exercise therapy, which are recommended within generalized fatigue management, are not considered suitable for patients with PEM [13]. Studies emphasize that rehabilitation strategies must be carefully planned. In this context, the pacing method emerges as a strategy that enables patients to carefully regulate their energy expenditure and prevent PEM attacks. This method requires patients to stay below a certain activity level and act according to their individual limits. Therefore, rehabilitation approaches should be individualized and symptom-based, and misguided exercise practices that could trigger PEM should be avoided [12].

The most frequently preferred referral clinic among physicians was physical medicine and rehabilitation (32.1%, n=34). This preference may be related to the fact that ME/CFS patients typically present with symptoms such as chronic fatigue, muscle pain, and functional limitations, which highlight the importance of physical rehabilitation. Psychiatry (21.7%, n=23) and neurology (20.8%, n=22) were also among the specialties to which referrals were frequently made. In contrast, referral rates to internal medicine (7.5%, n=8) and rheumatology (5.7%, n=6) were considerably lower (Table 3). Since internal medicine and rheumatology are disciplines primarily involved in diagnostic evaluations, the inherent uncertainty in the diagnostic process of ME/CFS given that the disease still lacks established biological markers might explain the lower preference for these departments. It appears that specialties focused on symptom management and rehabilitation (physical medicine and rehabilitation, psychiatry, and neurology) are more commonly chosen for referrals.

Notably, 12.3% (n=13) of the physicians stated that they do not prefer referring patients to another specialty, which is striking. When evaluating which awareness and attitude characteristics are associated with the reluctance to refer patients to other specialties, it was found that those who consider it appropriate for a patient with widespread body pain and fatigue to seek consultation at a pain clinic are less likely to prefer referrals to other specialties (Table 3; chi-square=11.405, p=0.044). This finding may suggest that some physicians prefer to manage ME/CFS within their own area of expertise or that there is no clear consensus regarding which specialty should follow up the disease. The interdisciplinary nature of ME/CFS and the absence of a standardized referral protocol may contribute to the varied attitudes among physicians. These findings indicate a potential lack of specific clinical guidelines. Therefore, enhancing interdisciplinary collaboration and developing standard protocols for referral processes are essential for the optimal management of ME/CFS.

Limitations

The questionnaire used in this study was of a pilot nature; therefore, comprehensive validity and reliability assessments were not conducted. However, our survey was designed as a questionnaire-based instrument to evaluate pain physicians’ knowledge and perceptions regarding ME/CFS, laying the groundwork for future validity and reliability studies. The physicians included in the study had completed their specialty training and, after meeting specific qualifications through a rigorous selection process, worked as pain subspecialists in highly specialized clinical fields. Consequently, the limited number of pain physicians working in this area restricts the sample size and limits the generalizability of the findings. Due to the cross-sectional design of the study, data collection was confined to a specific time frame, which does not allow for monitoring long-term changes or improvements in awareness levels.

## Conclusions

Conducting a comprehensive assessment of a patient's widespread pain and fatigue is essential for expanding the differential diagnosis, refining clinical perspectives, and considering a broader spectrum of potential conditions, particularly in cases where establishing a definitive diagnosis remains challenging. Given the evolving nature of research in this field, the definitions and diagnostic criteria for medically unexplained symptoms and syndromes are expected to become more precise in the coming years. This study, conducted among physicians specializing in pain management, offers valuable insights into their diagnostic reasoning, clinical decision-making, and awareness of ME/CFS. By highlighting the perspective of pain specialists, it provides a comparative framework that may inform clinical practice across multiple medical disciplines, potentially facilitating earlier recognition and improved management of ME/CFS. Furthermore, integrating a more structured approach to psychosocial factors, sleep disturbances, post-exertional malaise, autonomic dysfunction, and patient heterogeneity may pave the way for more personalized and effective treatment strategies. Incorporating standardized assessment tools for these variables in clinical settings could enhance diagnostic accuracy and patient outcomes. Future large-scale, multicenter studies incorporating objective biomarkers, neuroimaging techniques, and longitudinal follow-ups are warranted to bridge existing knowledge gaps, refine diagnostic criteria, and optimize both diagnostic and therapeutic pathways. Additionally, interdisciplinary collaborations between pain medicine, neurology, rheumatology, and psychiatry may provide a more holistic framework for managing complex conditions such as ME/CFS.
